# MicroRNAs: Not “Fine-Tuners” but Key Regulators of Neuronal Development and Function

**DOI:** 10.3389/fneur.2015.00245

**Published:** 2015-11-24

**Authors:** Gregory M. Davis, Matilda A. Haas, Roger Pocock

**Affiliations:** ^1^Development and Stem Cells Program, Monash Biomedicine Discovery Institute, Department of Anatomy and Developmental Biology, Monash University, Melbourne, VIC, Australia

**Keywords:** microRNA, brain, neurogenesis, axon guidance

## Abstract

MicroRNAs (miRNAs) are a class of short non-coding RNAs that operate as prominent post-transcriptional regulators of eukaryotic gene expression. miRNAs are abundantly expressed in the brain of most animals and exert diverse roles. The anatomical and functional complexity of the brain requires the precise coordination of multilayered gene regulatory networks. The flexibility, speed, and reversibility of miRNA function provide precise temporal and spatial gene regulatory capabilities that are crucial for the correct functioning of the brain. Studies have shown that the underlying molecular mechanisms controlled by miRNAs in the nervous systems of invertebrate and vertebrate models are remarkably conserved in humans. We endeavor to provide insight into the roles of miRNAs in the nervous systems of these model organisms and discuss how such information may be used to inform regarding diseases of the human brain.

## Introduction

MicroRNAs (miRNAs) are non-coding RNA molecules with a length of approximately 22 nucleotides, which act as post-transcriptional regulators of gene expression (1–4). Discovered just over two decades ago, miRNAs have been found to be abundant in most organisms and critically important for post-transcriptional control of mRNAs by regulating a predicted 60% of protein-coding genes (5). Prior to the discovery of the miRNA pathway, the *lin-14* gene in *Caenorhabditis elegans* was shown to be regulated by a 22-nucleotide partially complementary strand of RNA called *lin-4* (6, 7). However, a mechanistic understanding of this process remained unclear until the *let-7* gene was shown to encode a complementary sequence of *lin-41* to regulate developmental timing (8). This led to a paradigm shift in how mRNA regulation was viewed, and further investigation demonstrated that the miRNA pathway was evolutionarily conserved in most eukaryotes (9). Since then miRNAs have been shown to be required for key biological processes, such as cell fate, differentiation, apoptosis, and tumor suppression (10–13).

**Figure 1:**
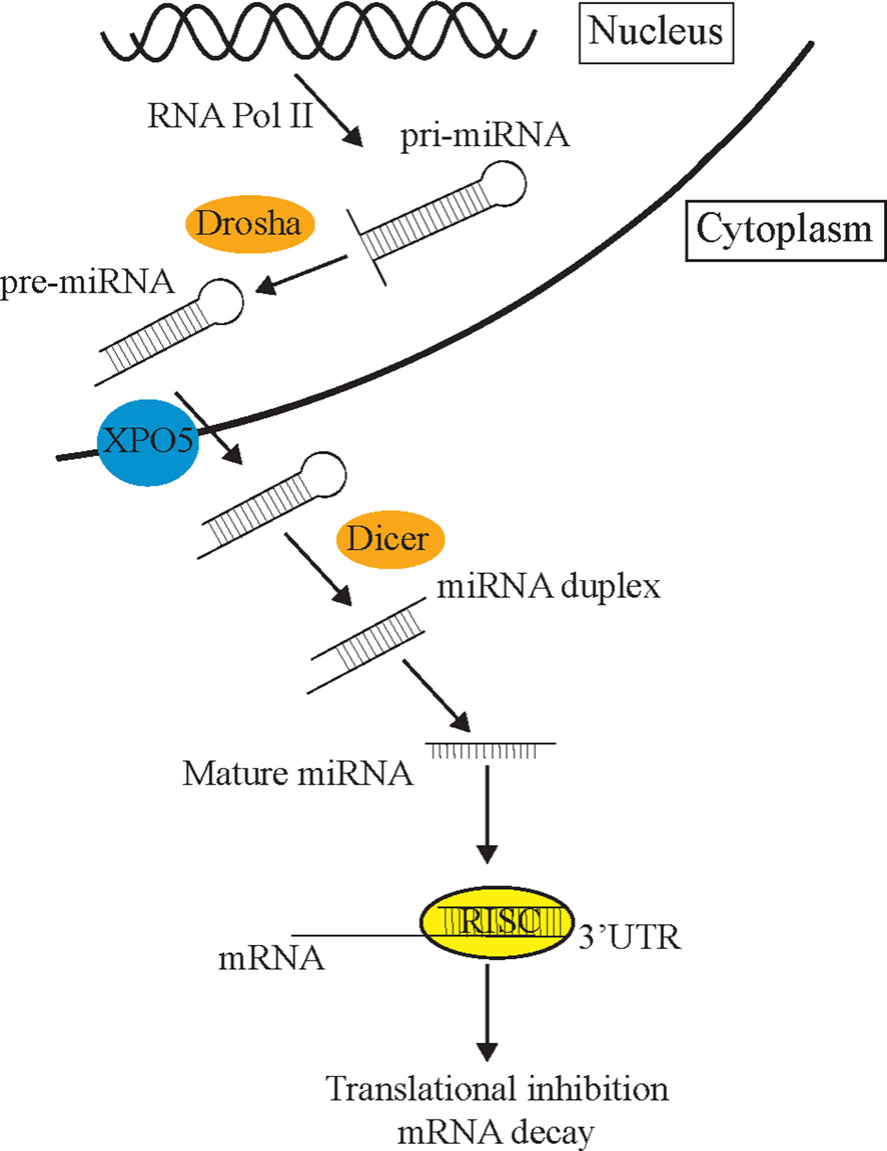
**The miRNA pathway**. Primary miRNAs (pri-miRNAs) are transcribed from the genome and form hairpin structures. Nuclear-localized Drosha endonuclease cleaves pri-miRNAs into approximately 70 nt precursor miRNAs (pre-miRNAs) which are then transported from the nucleus to the cytoplasm by Exportin-5 (XPO5) via the nuclear pore complex, where they are further cleaved by Dicer into mature 21–23 nt miRNA fragments. Once the strands separate, the guide strand is loaded into the RISC complex (AGO and different cofactors) to scan the transcriptome for partial complementary target transcripts. These sequences are either repressed by the RISC complex or degraded in P-bodies.

The process of miRNA biogenesis in animals can be briefly simplified into three fundamental steps (Figure 1) [for detailed review, see Ref. (14)]. First, double-stranded primary miRNA (pri-miRNA) short hairpin structures are transcribed by RNA polymerase II. Secondly, a nuclear-localized RNA endonuclease III, Drosha, defines one end of the pri-miRNA duplex and cleaves double-stranded RNA (dsRNA) transcripts into approximately 70 nt stem loops called precursor mRNAs (pre-miRNAs) (15). These pre-miRNAs are exported to the cytoplasm by Exportin-5 (XPO5) (16) where the Dicer enzyme cleaves pre-miRNA sequences into 21–23 nt mature miRNA double-stranded duplexes (17). Such miRNA duplexes load into a pre-RISC (pre-miRNA-induced silencing complex) which is a complex of Argonaute (AGO) and other proteins (18). Within the pre-miRISC, the “passenger” strand is removed leaving just the “guide” strand in the mature miRISC. The guide strand is normally the strand with a more thermodynamically unstable 5′ end (19). The released passenger strand is either degraded or loaded into a different miRISC complex to regulate a different group of target transcripts to the guide strand. The miRISC complexes then scan the transcriptome for partially complementary mRNA sequences. The miRNA then associates with a target mRNA by imperfect base-pairing, on the most part, to its 3′UTR and mediates post-transcriptional repression (PTR) or decay of specific mRNA targets (17, 20). The partially complementary sequences of miRNAs allow them to recognize and inhibit the expression of multiple mRNA transcripts (21). mRNA recognition is primarily determined through nucleotides 2–7 of the 5′ end or “seed” region of miRNAs (3). miRNAs can also initiate mRNA degradation by recruiting the mRNA degradation machinery, or through the use of cytoplasmic RNA granules known as Processing bodies (P-bodies), which can degrade mRNAs via cap removal and 5′–3′ exonuclease activity (22).

As mentioned above, the nature of miRNA targeting through imperfect complementarity means that single miRNAs have the potential to regulate the expression of hundreds of genes (3). In addition, certain genes have multiple miRNA-binding sites in their 3′UTRs and, therefore, multiple miRNA families potentially control their expression (3). 3′UTR length is often a determining factor as to its propensity to miRNA regulation (3). Such complex relationships between miRNAs and their targets enable exquisite control of gene regulatory networks. A better understanding of miRNA function in the control of such gene regulatory networks has been accelerated by the use of simple model organisms, such as *C. elegans* and *Drosophila*. Studies using these models are aided by their genetic amenability, short lifespans, and compact genomes. However, there are multiple mammalian-specific miRNAs for which the use of higher eukaryotes is required to study their biology.

### Functions of miRNAs in the Nervous System

The human brain contains approximately 86 billion neurons and trillions of synaptic connections (23). This complex organ is an integration center where environmental information is processed and used to make an appropriate action or decision. To effect brain function as a whole, neurons are organized into circuits which communicate with each other through rapidly acting synaptic connections and slower acting neuropeptide release. An inability to regulate these molecular communication processes is causative in developmental disorders, such as autism and schizophrenia, in addition to age-related decline of brain function (24, 25). Therefore, using model organisms to dissect these mechanisms at a molecular, anatomical and functional level will provide a greater understanding of neuronal-based disease.

**Figure 2:**
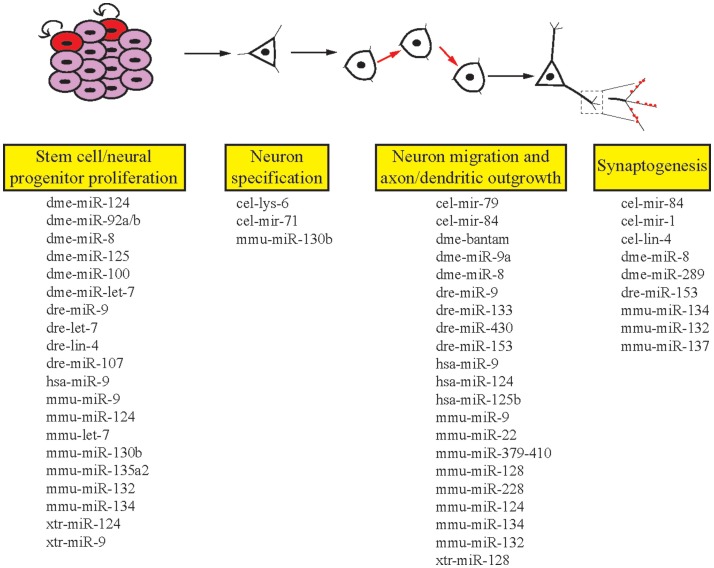
**Roles of miRNAs in different stages of neuronal development**. miRNAs are involved in the multiple stages of neuronal development in invertebrates and vertebrates. Listed here are the miRNAs we cover in this review that regulate single or multiple stages of neuronal development. cel, *Caenorhabditis elegans*; dme, *Drosophila melanogaster*; dre, *Danio rerio*; hsa, *Homo sapiens*; mmu, *Mus musculus*; xtr, *Xenopus tropicalis*.

The ability of the nervous system to adapt to different environmental conditions and stimuli requires a well-conserved and flexible repertoire of molecular mechanisms. miRNAs offer genetic networks’ additional layers of regulatory control and are abundantly expressed in all human tissues, including the brain (26). In addition to this, many miRNAs display specific temporal and spatial patterns of expression (27). Due to the high degree of complexity of the human brain, in addition to ethical concerns, deep mechanistic understanding of how miRNAs influence neurodevelopmental and functional processes has come from model organisms. This review aims to provide examples that reveal the important roles of miRNAs in the development and function of the nervous system (Figure 2). We focus on the crucial role model organism research has played in this area to provide insight into the functions of miRNAs.

## miRNA Regulation of the Nervous System in Invertebrates

### *Caenorhabditis* *elegans*

The initial discovery of miRNAs was made in the nematode *C. elegans* and, since that time, many fundamental studies implicating miRNAs in gene regulatory networks significant to neurodevelopment have been achieved using this model (28–30). *C. elegans* has proven demonstrative as many miRNAs are highly conserved throughout evolution, along with other well-known advantages of the model organism including its well-defined neuronal lineage map, neuroanatomy, and neural networks (31–35).

MicroRNAs have been shown to participate in instructing cell fate decisions made during the development of the nervous system of *C. elegans*. For example, the ASE neurons are a pair of morphologically similar, asymmetric gustatory sensory neurons, which have different roles: ASEL senses sodium, while ASER senses chloride (36). The neuron pair is derived from different lineages, which diverge at the four-cell stage of embryogenesis. A complex gene regulatory pathway, in which the miRNA *lsy-6* plays a central role, essentially achieves the specification of this pair of neurons during development. The Nkx homeobox transcription factor COG-1, which induces ASER fate, is inhibited by *lsy-6* in ASEL (37–39). It was subsequently shown that *lsy-6* expression itself is controlled by a complex mechanism involving two regulatory elements, firstly where transcription factors TBX-37/38 “prime” the *lsy-6* locus for expression by altering chromatin to an open state. Expression is then “boosted” by the transcription factor CHE-1, and *lsy-6* induces gene expression changes associated with asymmetrical generation of the ASEL and ASER neurons (40). This “priming” and “boosting” of *lsy-6* begins several cell divisions prior to the specification of ASEL neuron and is the first asymmetrically expressed gene in the ASEL and ASER neurons. This mechanism resulting in bilateral symmetry in the nervous system may provide important insights into how symmetry is established in the mammalian brain.

*mir-71* is another miRNA involved in neuron specification in *C. elegans*. *mir-71* controls cellular responses to calcium to specify asymmetry in function of the morphologically symmetrical AWC^ON^ and AWC^OFF^ olfactory neurons (41). *mir-71* controls this signaling pathway as it is expressed at a higher level in one neuron of the pair, which negatively regulates the calcium adaptor protein TIR-1/Sarm1 downstream of *nsy-4*/claudin and *nsy-5*/innexin, to specify the AWC^ON^ neuron (41). Interestingly, for this pair of neurons, cell fate is not rigid. That is, whether the left or right neuron is specified AWC^ON^ is not fixed, and if the AWC^ON^ is lost, AWC^OFF^ can convert to AWC^ON^ (41).

Aside from neuronal specification, evidence from *C. elegans* indicates that miRNAs are involved in gene regulation to control neuron migration and differentiation. It was demonstrated that a regulatory pathway essential for normal neuron migration and axon guidance involves *mir-79* (an ortholog of mammalian *miR-9*) (42). In *C. elegans*, loss of *mir-79* from epidermal cells caused an increase in expression of SQV-5 (a chondroitin synthase) and SQV-7 (a UDP-sugar transporter), both required for the biosynthesis of glycosaminoglycan (GAG) chains that are attached to proteoglycans (43). Defective regulation of SQV-5 and SQV-7 in the epidermis caused striking defects in the migration of hermaphrodite-specific neurons (HSNs). RNAi knockdown of *sqv-5* and *sqv-7* in *mir-79* deletion mutant animals restored the incidence of HSN defects to background levels. Additional work showed that this mechanism is required to regulate the addition of heparan sulfate chains on a glypican called LON-2. This work, therefore, defined a pathway through which *mir-79* expression in epidermal cells non-cell autonomously controls HSN migration (42).

Netrin-mediated axon guidance is also influenced by miRNA expression in *C. elegans* to ensure the correct timing of axon termination. Cell autonomous expression of *lin-4* (homologous with *miR-125a/b*) targets the transcription factor *lin-14* at completion of extension of the anterior ventral microtubule (AVM) axon, to inhibit netrin-mediated axon attraction (44). Such temporal loss of responsiveness is due to decreased expression of the netrin receptor UNC-40 through *lin-4*-mediated reduction of LIN-14. The cell-autonomous regulation of *lin-14* by *lin-4* is also required for the timing of axon extension of the HSNs (29). In *lin-4* loss of function animals, the HSNs do not extend their axons prior to the larval-adult transition, and adult axons have defective morphology. Therefore, *lin-4* is required to temporally regulate extension of axons in two neuronal paradigms in *C. elegans* (29).

Later in neuronal development, miRNAs also control synaptogenesis and remodeling in *C. elegans*. For example, *lin-4* targets *lin-14* to remodel motor neuron synapses during the first larval stage L1, when motor neurons eliminate their synapses with ventral muscles and instead form connections with dorsal muscles. The timing of this change is regulated by the heterochronic genes *lin-4*–*lin-14* (28) and *mir-84*–*hbl-1* (45). *mir-1* also plays important roles in synaptic function, by targeting the transcription factor MEF-2 to control neurotransmitter release at the neuromuscular junction (NMJ) (46). *mir-1* also targets synaptic proteins neuroligin and neurexin (47), which in humans are two synaptic proteins that have been linked with defects in synaptic function associated with autism spectrum disorders (ASDs) (48). Elucidating these mechanisms in *C. elegans* may reveal conserved pathways and provide important insights into human development and mechanisms underlying neurodevelopmental disorders.

Interestingly, miRNAs have also been implicated in the ­developmental decline of regenerative ability of the nervous system. The miRNA *let-7* is only expressed very weakly when initial axonogenesis occurs, and onset of expression with age contributes to a reduced capacity for regeneration of the AVM neurons (30). This mechanism involves a developmentally regulated loop including the TRIM protein LIN-41, among other factors. The exciting discovery that suppression of *let-7* could restore regenerative capacity (30) could be conserved in vertebrates, and indeed it has since been shown that suppression of Let-7 in primary cultured rat peripheral neurons increases their regenerative response (49).

Finally, the miRNA *mir-71* was found to regulate the physiology of *C. elegans* non-cell autonomously from the nervous system (50). The authors showed that *mir-71* expression in the AB lineage (nearly all neurons) is necessary and sufficient for lifespan extension of animals lacking a germline (50). The authors showed that neuronal *mir-71* regulates the localization and activity of the FOXO transcription factor DAF-16 in the intestine which acts downstream of insulin-like signaling to regulate metabolism and stress responses (50–52). Such non-cell autonomous regulation of intestinal DAF-16 by *mir-71* is via an, as yet, unidentified neuronal factor.

### *Drosophila* *melanogaster*

MicroRNAs in the fruit fly, *Drosophila melanogaster*, have been extensively investigated in various developmental processes [reviewed in Ref. (53)]. miRNAs are critical for all aspects of neuronal development, from regulating neural stem cells to regulating the events that occur at the NMJ. For example, control of neuronal progenitor proliferation is fine-tuned by the highly conserved miRNA, *miR-124*, which has been shown in various organisms to regulate neuronal stem cells (54–57). In *Drosophila*, *mir-124* targets *anachronism* (*ana*), an inhibitor of neuroblast proliferation. The absence of *miR-124* results in decreased proliferative activity, which is coupled with an increase in *ana* expression (58). In addition to this, *miR-124* is required for optimal regulation of dendrite growth and targets components of the retrograde BMP signaling pathway to regulate synaptic release at the NMJ (59).

Additional miRNAs that contribute to neuronal proliferation are the fly homologs of mammalian miR-92 and miR-200 – *miR-92a/b* and *miR-8*, respectively (60, 61). *miR-92a* is located in the intron and *miR-92b* in the 3′UTR of a putative DNA-binding protein, *jigr1*, and they suppress this host gene to regulate neural stem cell development to prevent premature differentiation (61). Additionally, *miR-8* has been implicated in regulating neuronal proliferation but is expressed in a glial cell population ensheathing the optic lobe neurepithelium (60). In the latter of these roles, *miR-8* is required for the temporal and spatial control of EGFR pathway ligand, Spitz, which controls accurate neuroepithelial proliferation and neuroblast formation (60).

*Drosophila* has been used as a model to identify miRNAs that temporally and spatially control neuronal differentiation and specification. An example of this is with olfactory neuronal morphogenesis, which is associated with accurate miRNA function. Loss of core components of the miRNA biogenesis machinery, including Pasha or Dicer, results in abnormal olfactory neuron morphogenesis (62). The basis for these defects was defined by studies of the mushroom body neurons (MB), which mediate olfactory responses and comprise of four invariant subtypes of neurons in various insects (63–66). The generation of MB neurons requires tight post-transcriptional regulation of the BTB-zinc finger *ch*ronologically *in*appropriate *mo*rphogenesis (*chinmo*) in postmitotic neurons (66). This is achieved by a group of miRNAs that are cotranscribed from a single locus and comprise of *miR-125*, *miR-100*, and the highly conserved *let-7*, collectively referred to as the *let-7-Complex* (*let-7-C*) (67). The initial discovery of *let-7* in *C. elegans* identified a heterochronic role for *let-7*, whereas in *Drosophila let-7* expression is not enriched in early development, but upregulation of *let-7-C* is associated with a downregulation of *chinmo* (68). This suggests that the mechanism identified in *C. elegans* where *let-7* regulates developmental timing functions in a different context in *Drosophila* to regulate the formation of MB neurons.

The involvement of miRNAs in dendrite growth is unclear, although a small number of studies in *Drosophila* have identified two miRNAs required for dendrite growth of sensory neurons. First, the miRNA, bantam, has been implicated in dendrite scaling by suppressing Akt kinase activity in nearby neurons and by regulating epithelial endoreplication (69, 70). Secondly, *miR-9a* (mammalian miR-9 homolog) acts from epithelial cells to fine-tune dendrite growth. This is achieved by regulating the activity of a cadherin-domain containing putative G-protein-coupled receptor, Fmi, which functions to suppress dendrite growth (71). *miR-9a* also acts with *miR-7* to control the development of sensory organs. In *Drosophila*, sensory organs develop from single organ precursor cells (SOPs), which are generated from clusters of cells expressing proneural genes. This process is temporally controlled by Notch signaling and two transcription factors that regulate proneural gene expression. These consist of Senseless, a positive regulator of SOP cells, which is targeted by *miR-9a*, and a negative regulator, Enhancer of Split, which is targeted by *miR-7* (72–74). Furthermore, *miR-9a* is also influenced by the RBA-binding protein, TDP-43, mutants of which display increased SOP cells coupled with decreased *miR-9a* expression (75). Although the exact requirement of TDP-43 in this process is unclear, genetic interaction assays suggest that SOP specification requires TDP-43 for accurate neuronal differentiation by influencing *miR-9a* activity.

MicroRNAs have also been shown to regulate the NMJ in *Drosophila* embryos and larvae. *miR-8* and *miR-289* are required to suppress activity-dependent synaptic growth by targeting genes involved in axon development and growth. *miR-8* downregulates *wingless*, a presynaptic regulatory protein required for activity-dependent axon terminal growth at the NMJ (76). In this context, *miR-8* regulates the timing of synaptic expansion to correlate with the growth of target muscles. Furthermore, *miR-8* regulates the embryonic expression of two synaptic immunoglobulin superfamily cell adhesion molecules (IgCAMs), Fasciclin III (FasIII), and Neuroglian (Nrg) (77). Taken together, these studies lay a foundation for further study into the role(s) of *miR-8* in presynaptic events, as well as the timing of synaptic assembly with neuron–muscle association.

Finally, *miR-8* has also been shown to regulate apoptosis in the CNS of *Drosophila* (78). *miR-8* regulates the expression of the transcriptional corepressor Atrophin to a particular threshold level. Loss of *miR-8* results in increased *atrophin* levels and apoptosis; however, reduction of *atrophin* expression below the threshold set by *miR-8* causes extra tissue being generated (78). Precise tuning of *atrophin* levels is, therefore, required to prevent neurodegeneration in the CNS of *Drosophila*.

## miRNA Regulation of the Nervous System in Vertebrates

### *Xenopus* *laevis*

Neuronal development and function have been extensively studied in the *Xenopus laevis* tadpole [reviewed in Ref. (79)]. However, in contrast to other model organisms, the influence of miRNAs during neurodevelopmental processes has received less attention. Nevertheless, certain conserved miRNAs investigated in *Xenopus* offer new insights to their function. For example, similar to other model organisms, miR-124 regulates early neurogenesis. However, in *Xenopus*, miR-124 is expressed from the beginning of eye development where it plays an important role in regulating retinal neurogenesis in the optic vesicle and forebrain (57, 80, 81). In addition to this, miR-129, miR-155, miR-214, and miR-222 contribute to developmental timing of retinal progenitor cells by regulating the activity of the transcription factors, *xotx2* and *xvsx1*, which are both required for promoting the late-stage progenitor cells to differentiate into bipolar neurons (82).

The highly conserved miR-9 is also required for neurogenesis along the anterior–posterior axis by targeting the transcription factor, *hairy1*, although its function varies from the hindbrain to the forebrain. In the forebrain, regulation of *hairy1* by miR-9 influences proliferation of neural progenitor cells through Fgf8 signaling, but via Wnt signaling in the hindbrain (83). This suggests positional specificity regarding miR-9 function. Defects associated with the nonsense-mediated mRNA decay (NMD) pathway result in neurological disorders in humans (84, 85). Interestingly, in *Xenopus*, miR-128 has been shown to repress NMD by targeting the RNA helicase, UPF1, and the exon-junction cofactor, MLN51 (86). This process allows upregulation of specific mRNAs required for differentiating neuronal cells, which are normally targeted by NMD. Moreover, this mechanism is highly conserved in mammals and represents a dual mRNA regulatory network to maintain neuron development and function (86).

### *Danio* *rerio*

*Danio rerio* (zebrafish) is a valuable model system that has been used to uncover neurodevelopmental functions for miRNAs, with the advantage that zygotic loss of miRNAs can be examined in the absence of maternal compensation mechanisms, since the zygote develops outside of the mother (87). Embryos carrying *dicer* mutations display severe developmental defects, including delayed embryogenesis, perturbed neurulation, and formation of brain ventricles, as well as ill-defined anatomical boundaries, such as the midbrain-hindbrain boundary (MHB) (87). However, interestingly the same study also showed that despite the gross morphological defects, gene expression, and neuron specification were maintained within patterned regions, such as the forebrain and hindbrain rhombomeres. Later stage neuronal differentiation, such as axon extension, was also severely affected by loss of *dicer* (87).

In zebrafish, miRNAs are expressed in neural cells throughout the different stages of development in addition to in mature neurons. They can have ubiquitous or cell-specific expression patterns. Many conserved miRNAs are expressed at the same developmental timepoints as other vertebrates, for example, *miR-9* and *let-7* are expressed in both proliferating and differentiating cells (88). As in *C. elegans*, *lin-28* and its downstream heterochronic genes *let-7* and *lin-4/miR-125b* are expressed during development to coordinate cell proliferation (89).

In zebrafish hindbrain development, *miR-107* stabilizes dicer levels, which maintains a specific level of *miR-9* biogenesis to regulate optimal proliferation of neural progenitors (90). *miR-9* inhibits proliferation at the MHB and hindbrain ventricular zone through targeting of proproliferation genes *her5*, *her6*/*Hes1*, and *zic5* and then later also influences neuronal maturation by regulating *elav3*/*HuC* (91–93). Additionally, *miR-9* overexpression causes a strong reduction in the MHB and cerebellum, as well as blurred somatic boundaries and altered cell fates, through downregulation of *fgfr1* in the Fgf signaling pathway (91).

More recently, zebrafish hindbrain development has been used as a model system to uncover precise mechanisms of the miRNA-mediated mRNA decay pathway (94). In this study, a genetic screen implicated *cnot8*, which was known to have deadenylase activity in polyA tail removal in mRNA turnover. Furthermore, the role of *cnot8* in the Fgf signaling pathway is responsible for hindbrain dopaminergic neuron differentiation, and application of a drug inhibiting Fgf signaling partially restored the mutant phenotype (94). Zebrafish studies also elucidated the mechanisms through which morphine influences dopaminergic neuron differentiation, since maternal influence can be removed from developmental events. Morphine downregulates *miR-133*, which increases *pitx3* expression thereby promoting dopaminergic neuron maturation (95). This study revealed important information relating to neural networks involved in drug addiction.

In the later stages of zebrafish neurodevelopment, *miR-430* controls trigeminal sensory neuron migration. These sensory neurons arise from the neural crest and placodes, and their migratory journey of up to 120 μm requires the chemokine SDF1a and its receptor Cxcr4b. The border of SDF1a expression shifts continually to make a tightly regulated chemotactic path for neurons to migrate, and *miR-430* is important for clearing SDF1a from the pathway that neurons have passed through (96).

Finally, in zebrafish embryos, miRNAs are also involved in dendritic spine formation and synaptogenesis. For example, knockdown of *miR-153* caused a sevenfold increase in spontaneous body movement, and the synaptic protein SNAP-25, which is involved in vesicular exocytosis, was found to be the target (97).

### *Mus* *musculus*

The earliest mammalian studies following the discovery of miRNAs in *C. elegans* quickly demonstrated the crucial nature of the class of non-protein-coding RNAs in mammals through the generation of mice carrying deletions for miRNA-processing pathway components including *Dicer*, *Dgcr8*, and *Argonaute*. Argonaute proteins are essential components of the RISC complex, facilitating translational inhibition or target mRNA cleavage, and *Argonaute 2* mutants show an early and severe neurodevelopmental phenotype, with neural tube closure failing to occur (98). Deletion of another component of the miRNA-processing pathway, *Dgcr8*, causes microcephaly in mutant mice but is much less severe than loss of Dicer mutants (99). Under investigation is the potential importance of Dgcr8 in Di George syndrome, a multifaceted disorder where 30 genes including *Dgcr8* are deleted, and has been associated with schizophrenia (100).

Loss of Dicer causes lethality in lower eukaryotes, such as *C. elegans* and *Drosophila* (101, 102). However, the neurodevelopmental consequence of *Dicer* loss in mice has been investigated in greater depth using conditional deletion mutants (103, 104). This led to further confirmation of both the spatial and temporal importance of miRNA-mediated pathways in multiple phases of mammalian CNS development. One study used conditional *Dicer* deletions generated with Emx-Cre (excitatory cerebral cortex neurons) and Nestin-Cre (all CNS neurons) mouse lines at different stages of embryonic cerebral cortex development showed that miRNAs are important for three major phases of cerebral cortex development: neuronal progenitor proliferation, neuronal migration, and differentiation (105).

Conditional mutants have been further used to confirm the importance of miRNAs in the progression of cerebral cortex development, with Cre-recombinase-driven deletion of *Dicer* under the control of Foxg1, Emx1, Nes, Nex, and CamkII promoters (106). These models have shown that in the early stages of cerebral cortex development, conditional deletion leads to a loss of neurons, either due to loss of the neural progenitor pool or increased apoptosis. Deletion of *Dicer* leads to neuron migration defects and impaired cellular differentiation, as well as cell fate changes and cortical lamination defects (106).

Conditional deletion of *Dicer* from the embryonic day 8 (E8) telencephalon causes a loss of radial glial progenitor markers, including nestin, Sox9, and ErbB2, which then results in an increase in basal progenitors and postmitotic neurons (107). Interestingly, increased apoptosis was also observed, and this was correlated with reduced expression of *miR-9* and *miR-124* (107), two miRNA families that have been widely implicated in brain development. *Dicer* conditionally deleted using the Nex-Cre promoter (targeting neurons of pyramidal fate) resulted in significantly smaller mouse brains, due to increased packing density of neurons, as well as abnormal neuron differentiation, but no defect in neuron production or cortical lamination (107). Conditional deletion of *Dicer* by α-CaMKII-Cre in the embryonic forebrain resulted in microcephaly due to increased apoptosis, rather than neuronal migration defects, since lamination appeared unaffected. Reduced dendritic branching and dendritic spine development was also observed (103), along with ataxia and reduced life span. Interestingly, *Dicer* deletion in neural crest cells (by Wnt1-Cre) does not affect migration and early differentiation, but miRNA pathways appear to be required for the survival of peripheral nervous system (PNS) neurons, because in *Dicer* deletion mutants PNS neurons are lost after completion of migration and differentiation due to apoptosis (108, 109). Thus, while *Dicer* mutants all point toward dysregulated nervous system development, individual studies have reported slightly different mechanisms. Whether this is due to the timing of deletion, the promoter driving Cre-recombinase or which miRNAs are being lost due to blocking the processing pathway is not clear.

*Dicer* deletions have been useful in determining the overall impact of loss of miRNAs on gene regulation, but more information has been gained from studying the roles of specific individual miRNAs. The *let-7* family, which was one of the first described in *C. elegans*, was subsequently also shown in the mouse to play a significant role in maintaining the balance in neuronal progenitor proliferation and neurogenesis, since TRIM32 (110) and SOX-2 (111) influence *let-7* levels to maintain cells in a proliferative state. *let-7b* in turn regulates neural stem cell proliferation by targeting the stem cell regulators TLX and cyclinD1 (112).

The *miR-9* family is one of the most highly conserved and abundantly expressed miRNA families in the vertebrate brain and is also involved in balancing neural progenitor proliferation and controlling progenitor state (93). *miR-9* regulates early progenitor proliferation in the mammalian brain through the transcription factors Hes1 (113), Foxg1, Elav2, Pax-6, as well as Gsh2 (114). Confirming its proproliferative role, loss of *miR-9* suppresses neural stem cell proliferation, through stathmin (115).

The *miR-124* family is also conserved from *C. elegans* to humans. It is expressed by differentiating neurons in the subventricular zone of the developing mouse cortex, where it controls apical/basal progenitor progression (56). It is also important for continual production of neurons in the subventricular zone of the adult mouse brain (116). In neuronal differentiation, *miR-124* is involved in a mechanism with the transcriptional repressor REST, whereby REST represses *miR-124a* and expression of neuronal genes in non-neuronal cells and neural progenitors. But at later stages, REST ceases to repress *miR-124a*, allowing non-neuronal transcript degradation and thus promoting neuronal differentiation (117). REST also has miR-124-binding sites in its 3′UTR, suggesting a complex regulatory loop exists (118). Another transcriptional repressor involved in the REST complex, MeCP2, also has predicted *miR-124*-binding sites in its 3′UTR (118), and MeCP2 mutation or copy number variant has been implicated in multiple neurodevelopmental disorders including X-linked intellectual disability and autism (119, 120).

Therefore, the above-mentioned *in vivo* studies suggest that *miR-9* and *miR-124* are major players in the regulation of cerebral cortex development, but *in vitro* studies have shown that *miR-9* and *miR-124* can drive the neurogenic program. When Yoo and colleagues (121) added *miR-9* and *miR-124* precursors to cultured neonatal foreskin fibroblasts, they were able to directly convert them to neurons expressing the mature marker MAP2, albeit at a conversion rate of <5%. Neuron conversion was improved remarkably by the addition of *NEUROD2*, *ASCL1*, and *MYT1L*, a combination that has previously been shown to produce neurons (122). Importantly, after 4 weeks in culture, the neurons expressed synaptic markers and displayed electrophysiological properties consistent with neurons (121). This exciting study suggests that miRNAs are not only involved in controlling gene expression in neurodevelopment, but could be considered among the master regulators of neurogenesis in mammalian cells.

Many other miRNAs have been implicated in the earliest stages of mammalian brain development and regulate important pathways in development and disease. For example, *miR-130b* has been shown to regulate *Fmr1* expression, which is lost in the disorder fragile X syndrome. *Fmr1* loss causes increased progenitor proliferation and altered neuronal fate specification (123). *miR-135a2* regulates Wnt signaling in midbrain dopaminergic neuron proliferation in a regulatory circuit with Lmx1b (124), and in the mouse cortex, *miR-134* promotes neural progenitor cell proliferation and counteracts apoptosis and differentiation (125). *miR-34*, another conserved miRNA, appears to be linked with neuron proliferation, because overexpression of *mir-34* in human stem cells suppressed the expression of 136 neuronal progenitor genes that possess putative *miR-34* target sites. Gene ontology showed that these genes are overwhelmingly involved in cell motility and energy production (126).

In addition to being crucial for neuronal progenitor proliferation, *miR-9* and *miR-124* are emerging as key regulators of neuron migration. *miR-9*, along with *miR-132*, represses Foxp2 to regulate radial migration in the developing mouse cortex. Ectopic expression of Foxp2 in the developing cortex was counteracted by increased endogenous expression of *miR-9* and *miR-132* (127). In a different mechanism, *miR-124* and *miR-22* regulate cell shape changes in migrating cortical neurons by controlling expression of doublecortin, a microtubule-associated cytoskeleton protein involved in cell shape remodeling through multipolar and bipolar phases in migrating neurons, through CoREST/REST (128). Recently, the *miR-379-410* cluster was shown to regulate N-cadherin expression, a crucial factor in maintaining tissue structure in the developing cortex. Overexpression of these miRNAs in radial glial cells decreased N-cadherin expression, causing increased stem cell differentiation and migration (129). *miR-128* in the brain regulates Phf6, which is a mutated gene in the disorder Borjeson–Forssman–Lehmann syndrome, and ectopic expression of *miR-228* in the developing brain leads to neuron migration defects, neurite outgrowth, and electrophysiological changes (130).

Specific miRNAs exhibit increased expression in the latter stages of nervous system development in the mouse and are implicated in final differentiation, neurite extension, and synapse formation. These miRNAs include *miR-134* (125, 131) and *miR-132* (132–134). Also, *miR-124* regulates RhoG, which is a major player in the control of axon and dendrite outgrowth and complexity, in mouse hippocampal neurons (135).

Very recently, expression of a *miR-137* gain-of-function construct, first in cell lines, and then in the mouse hippocampal dentate gyrus, was shown to downregulate three well-known presynaptic proteins: complexin-1 (Cplx1), *N*-ethylmaleimide-sensitive fusion protein (Nsf), and synaptotagmin-1 (Syt1) (136). *In vivo*, this was accompanied by fewer synaptic vesicles and impaired hippocampal LTP, and impaired hippocampal dependent learning in behavioral testing. Excitingly, some of these defects were rescued by codelivery of a *miR-137* sponge construct, which sequestered endogenous *miR-137*. These findings and rescue in the mouse brain are particularly promising, given the vast data implicating *miR-137* SNPs in schizophrenia (136).

### *Homo* *sapiens*

An estimated 70% of all miRNAs are expressed in the human nervous system [reviewed in Ref. (137)], although only a small number of miRNAs appear to be regulated during neuronal differentiation (138). Moreover, temporal and spatial distribution of miRNAs in human donor brains, as well as target genes associated with neurodevelopmental diseases have been identified (139). Despite this, ethical complications prevent in-depth mechanistic studies from occurring in humans. Therefore, expression of these conserved miRNAs, coupled with mechanistic studies from model organisms or cell lines, has allowed for neuronal miRNAs to be understood in greater detail [for reviews, see Ref. (140, 141)]. The majority of investigations in humans use cell lines or use screening-based approaches for miRNAs that are associated with populations of particular neurodevelopmental disease states. Cell line-based approaches, despite their *in vitro* limitations have characterized several miRNAs required for neuronal proliferation and differentiation. For example, overexpression of the highly conserved miR-9 promotes proliferation of neural progenitor cells in human embryonic stem cells (115). miR-9 along with miR-124 and miR-125b has also been associated with inducing human pluripotent stem cells to differentiate into neurons (142). Although described previously in *Drosophila* and in the mouse, miR-9 is a good example of an evolutionary conserved miRNA that contributes to various aspects of neuronal development.

Aberrant expression of miRNAs has been associated with different neurodevelopmental disorders, such as schizophrenia, autism, Down syndrome, fragile X syndrome, and Rett syndrome [reviewed in Ref. (143–145)]. Determining if the aberrant expression of all of these associated miRNAs is simply the consequence of abnormal neuronal development or the cause of the disorder itself is challenging. Nonetheless, several studies have identified numerous aberrantly expressed miRNAs associated with bipolar and schizophrenic patients (146, 147). For example, a contributing factor in schizophrenia is decreased function of the *N*-methyl-d-aspartate (NMDA) receptor and delivery of NMDA receptor antagonists phenocopy the conditions associated with schizophrenia (148–150). Interestingly, miR-132 is downregulated in schizophrenic patients and has also been shown to contribute to the depolarization of the NMDA receptor (151, 152), suggesting that miR-132 may be a candidate for potential therapeutics. Defects associated not only with the expression of key miRNAs, but also at a genetic level have been implicated in schizophrenia. A genome-wide study of over 40,000 schizophrenic patients identified a SNP within the putative coding region of miR-137 resulting in decreased efficiency of miR-137 function (153). This is further supported by additional studies that have shown variation of miR-137 affects brain activation and function (154–156). Unlike examples of differentially expressed miRNAs, miR-137 suggests a direct genetic-miRNA association with schizophrenia.

Neurological diseases that give rise to ASDs, such as fragile X syndrome and Rett syndrome, display elevated and depleted miRNA expression [for detailed review, see Ref. (157)]. In addition to this, mutations associated with the miRNA machinery are thought to contribute to the progression of ASDs. For example, mutations in the RNA-binding protein, fragile X mental retardation 1 protein (FMR1), are associated with fragile X syndrome patients (158). In *Drosophila*, FMR1 functions as a RISC cofactor to maintain miRNA function (159). Another example is associated with Rett syndrome, which is caused by mutations associated with the methyl-CpG-binding protein 2 (MECP2) (160), which prevent nuclear miRNA processing by regulating the Drosha complex (161). Despite the importance of these proteins in human disease states, most mechanistic insights regarding FMR1 and MECP2 have come from investigations employing model organisms. Furthermore, these FMR1 and MECP2 studies show how mutations in key proteins associated with miRNA targeting or processing contribute to neurological diseases that give rise to autism.

Screening-based approaches to understand ASD-associated miRNAs involve postmortem analysis of autistic patients, as well as assessing circulating miRNAs in serum and plasma. This screening approach not only has led to a greater understanding of miRNAs associated with ASDs but also has potential to use selected miRNAs as non-invasive biomarkers for ASDs. Postmortem analysis of the cerebellar cortex identified 28 dysregulated miRNAs from 13 autism patients (162). This included miRNAs that are predicted to target the synaptic scaffolding protein, SHANK3, and the presynaptic cell adhesion protein, NRXN1, which are both associated with ASDs (163, 164). More recently, two studies carried out in live patients have identified 13 miRNAs in 55 children, as well as five miRNAs in 15 patients in China that are differentially expressed in serum and plasma in children with ASDs (165, 166). These investigations have also revealed the enrichment of predicted target genes of these differentially expressed miRNAs in various neurological pathways, suggesting a potential use for diagnosis and future therapeutic approaches.

## Conclusion

Since the initial discovery of miRNAs being involved in developmental timing of larval development in *C. elegans* (6, 8, 9), small non-coding RNAs have been implicated in a multitude of biological processes. *lin-4* and *let-7* were identified in unbiased forward genetic screens, as was *lsy-6*, the first miRNA shown to be involved in the nervous system (38). These genetic approaches enabled the identification of miRNAs with very specific roles during development. As such, these particular functions may have been overlooked when using reverse genetic techniques. However, waiting for such chance discoveries takes much time; therefore, other approaches are required to systematically drive miRNA discoveries forward. With the advent of temporal expression pattern analysis, sophisticated RNA sequencing and proteomic approaches, and miRNA prediction algorithms, the ability to move from a mutation in a specific miRNA to phenotype is a possibility using model organisms. Therefore, the meticulous scrutiny of miRNA expression patterns and screening for anatomical, functional, and behavioral phenotypes has proved fertile ground in the identification of roles for miRNAs in the brain.

In humans, the expression of a number of miRNAs has been correlated with neurodevelopmental disorders (143–145); however, the molecular mechanisms through which they act are not understood. Using model organisms as a discovery tool enables researchers to study the function of these miRNAs in depth. Due to the high degree of conservation between model organisms and humans, the elucidation of molecular mechanisms that control neuronal development using these models will help identify novel therapeutic approaches in the future.

## Conflict of Interest Statement

The authors declare that the research was conducted in the absence of any commercial or financial relationships that could be construed as a potential conflict of interest.

## References

[1] Bartel DP MicroRNAs. Genomics, biogenesis, mechanism, and function. Cell (2004) 116:281–97. 10.1016/S0092-8674(04)00045-5 14744438

[2] Bartel DP Chen CZ Micromanagers of gene expression: the potentially widespread influence of metazoan microRNAs. Nat Rev Genet (2004) 5:396–400. 10.1038/nrg1328 15143321

[3] Bartel DP. MicroRNAs: target recognition and regulatory functions. Cell (2009) 136:215–33. 10.1016/j.cell.2009.01.002 19167326 PMC3794896

[4] Ebert MS Sharp PA. Roles for microRNAs in conferring robustness to biological processes. Cell (2012) 149:515–24. 10.1016/j.cell.2012.04.005 22541426 PMC3351105

[5] Grun D Wang YL Langenberger D Gunsalus KC Rajewsky N. microRNA target predictions across seven Drosophila species and comparison to mammalian targets. PLoS Comput Biol (2005) 1:e13. 10.1371/journal.pcbi.0010013 16103902 PMC1183519

[6] Lee RC Feinbaum RL Ambros V. The *C. elegans* heterochronic gene lin-4 encodes small RNAs with antisense complementarity to lin-14. Cell (1993) 75:843–54. 10.1016/0092-8674(93)90529-Y 8252621

[7] Wightman B Ha I Ruvkun G. Posttranscriptional regulation of the heterochronic gene lin-14 by lin-4 mediates temporal pattern formation in *C. elegans*. Cell (1993) 75:855–62. 10.1016/0092-8674(93)90530-4 8252622

[8] Reinhart BJ Slack FJ Basson M Pasquinelli AE Bettinger JC Rougvie AE The 21-nucleotide let-7 RNA regulates developmental timing in *Caenorhabditis elegans*. Nature (2000) 403:901–6. 10.1038/35002607 10706289

[9] Pasquinelli AE Reinhart BJ Slack F Martindale MQ Kuroda MI Maller B Conservation of the sequence and temporal expression of let-7 heterochronic regulatory RNA. Nature (2000) 408:86–9. 10.1038/35040556 11081512

[10] Houbaviy HB Murray MF Sharp PA. Embryonic stem cell-specific MicroRNAs. Dev Cell (2003) 5:351–8. 10.1016/S1534-5807(03)00227-2 12919684

[11] Xu P Vernooy SY Guo M Hay BA. The Drosophila microRNA Mir-14 suppresses cell death and is required for normal fat metabolism. Curr Biol (2003) 13:790–5. 10.1016/S0960-9822(03)00250-1 12725740

[12] Lu J Getz G Miska EA Alvarez-Saavedra E Lamb J Peck D MicroRNA expression profiles classify human cancers. Nature (2005) 435:834–8. 10.1038/nature03702 15944708

[13] Rosa A Spagnoli FM Brivanlou AH. The miR-430/427/302 family controls mesendodermal fate specification via species-specific target selection. Dev Cell (2009) 16:517–27. 10.1016/j.devcel.2009.02.007 19386261

[14] Winter J Jung S Keller S Gregory RI Diederichs S. Many roads to maturity: microRNA biogenesis pathways and their regulation. Nat Cell Biol (2009) 11:228–34. 10.1038/ncb0309-228 19255566

[15] Lee Y Ahn C Han J Choi H Kim J Yim J The nuclear RNase III Drosha initiates microRNA processing. Nature (2003) 425:415–9. 10.1038/nature01957 14508493

[16] Yi R Qin Y Macara IG Cullen BR. Exportin-5 mediates the nuclear export of pre-microRNAs and short hairpin RNAs. Genes Dev (2003) 17:3011–6. 10.1101/gad.1158803 14681208 PMC305252

[17] Lee Y Jeon K Lee JT Kim S Kim VN. MicroRNA maturation: stepwise processing and subcellular localization. EMBO J (2002) 21:4663–70. 10.1093/emboj/cdf476 12198168 PMC126204

[18] Krol J Loedige I Filipowicz W. The widespread regulation of microRNA biogenesis, function and decay. Nat Rev Genet (2010) 11:597–610. 10.1038/nrg2843 20661255

[19] Khvorova A Reynolds A Jayasena SD. Functional siRNAs and miRNAs exhibit strand bias. Cell (2003) 115:209–16. 10.1016/S0092-8674(03)00801-8 14567918

[20] Pasquinelli AE. MicroRNAs and their targets: recognition, regulation and an emerging reciprocal relationship. Nat Rev Genet (2012) 13:271–82. 10.1038/nrg3162 22411466

[21] Carroll AP Goodall GJ Liu B. Understanding principles of miRNA target recognition and function through integrated biological and bioinformatics approaches. Wiley Interdiscip Rev RNA (2014) 5:361–79. 10.1002/wrna.1217 24459110

[22] Jain S Parker R The discovery and analysis of P Bodies. Adv Exp Med Biol (2013) 768:23–43. 10.1007/978-1-4614-5107-5_3 23224963

[23] Azevedo FA Carvalho LR Grinberg LT Farfel JM Ferretti RE Leite RE Equal numbers of neuronal and nonneuronal cells make the human brain an isometrically scaled-up primate brain. J Comp Neurol (2009) 513:532–41. 10.1002/cne.21974 19226510

[24] Nyberg L Lovden M Riklund K Lindenberger U Backman L. Memory aging and brain maintenance. Trends Cogn Sci (2012) 16:292–305. 10.1016/j.tics.2012.04.005 22542563

[25] Penzes P Buonanno A Passafaro M Sala C Sweet RA. Developmental vulnerability of synapses and circuits associated with neuropsychiatric disorders. J Neurochem (2013) 126:165–82. 10.1111/jnc.12261 23574039 PMC3700683

[26] Narayan A Bommakanti A Patel AA. High-throughput RNA profiling via up-front sample parallelization. Nat Methods (2015) 12:343–6. 10.1038/nmeth.3311 25730493 PMC4451056

[27] Chen W Qin C. General hallmarks of microRNAs in brain evolution and development. RNA Biol (2015) 12:701–8. 10.1080/15476286.2015.1048954 26000728 PMC4615839

[28] Hallam SJ Jin Y. lin-14 regulates the timing of synaptic remodelling in *Caenorhabditis elegans*. Nature (1998) 395:78–82. 10.1038/25757 9738501

[29] Olsson-Carter K Slack FJ. A developmental timing switch promotes axon outgrowth independent of known guidance receptors. PLoS Genet (2010) 6. 10.1371/journal.pgen.1001054 20700435 PMC2916846

[30] Zou Y Chiu H Zinovyeva A Ambros V Chuang CF Chang C. Developmental decline in neuronal regeneration by the progressive change of two intrinsic timers. Science (2013) 340:372–6. 10.1126/science.1231321 23599497 PMC4074024

[31] Sulston JE. Post-embryonic development in the ventral cord of *Caenorhabditis elegans*. Philos Trans R Soc Lond B Biol Sci (1976) 275:287–97. 10.1098/rstb.1976.0084 8804

[32] Sulston JE Horvitz HR Post-embryonic cell lineages of the nematode, *Caenorhabditis elegans*. Dev Biol (1977) 56:110–56. 10.1016/0012-1606(77)90158-0 838129

[33] Sulston JE Neuronal cell lineages in the nematode *Caenorhabditis elegans*. Cold Spring Harb Symp Quant Biol (1983) 48(Pt 2):443–52. 10.1101/SQB.1983.048.01.049 6586366

[34] Sulston JE Schierenberg E White JG Thomson JN. The embryonic cell lineage of the nematode *Caenorhabditis elegans*. Dev Biol (1983) 100:64–119. 10.1016/0012-1606(83)90201-4 6684600

[35] Durbin R.M Studies on the Development and Organisation of the Nervous System of Caenorhabditis Elegans. Ph.D. thesis, Cambridge: University of Cambridge (1987).

[36] Bargmann CI Horvitz HR. Chemosensory neurons with overlapping functions direct chemotaxis to multiple chemicals in *C. elegans*. Neuron (1991) 7:729–42. 10.1016/0896-6273(91)90276-6 1660283

[37] Chang S Johnston RJ Jr Hobert O. A transcriptional regulatory cascade that controls left/right asymmetry in chemosensory neurons of *C. elegans*. Genes Dev (2003) 17:2123–37. 10.1101/gad.1117903 12952888 PMC196454

[38] Johnston RJ Hobert O. A microRNA controlling left/right neuronal asymmetry in *Caenorhabditis elegans*. Nature (2003) 426:845–9. 10.1038/nature02255 14685240

[39] Sarin S Antonio C Tursun B Hobert O. The *C. elegans* Tailless/TLX transcription factor nhr-67 controls neuronal identity and left/right asymmetric fate diversification. Development (2009) 36:2933–44. 10.1242/dev.040204 19641012 PMC2723065

[40] Cochella L Hobert O. Embryonic priming of a miRNA locus predetermines postmitotic neuronal left/right asymmetry in *C. elegans*. Cell (2012) 151:1229–42. 10.1016/j.cell.2012.10.049 23201143 PMC3529140

[41] Hsieh YW Chang C Chuang CF. The microRNA mir-71 inhibits calcium signaling by targeting the TIR-1/Sarm1 adaptor protein to control stochastic L/R neuronal asymmetry in *C. elegans*. PLoS Genet (2012) 8:e1002864. 10.1371/journal.pgen.1002864 22876200 PMC3410857

[42] Pedersen ME Snieckute G Kagias K Nehammer C Multhaupt HA Couchman JR An epidermal microRNA regulates neuronal migration through control of the cellular glycosylation state. Science (2013) 341:1404–8. 10.1126/science.1242528 24052309

[43] Bulik DA Robbins PW. The *Caenorhabditis elegans* sqv genes and functions of proteoglycans in development. Biochim Biophys Acta (2002) 1573:247–57. 10.1016/S0304-4165(02)00391-4 12417407

[44] Zou Y Chiu H Domenger D Chuang CF Chang C. The lin-4 microRNA targets the LIN-14 transcription factor to inhibit netrin-mediated axon attraction. Sci Signal (2012) 5:ra43. 10.1126/scisignal.2002437 22692424 PMC3670680

[45] Thompson-Peer KL Bai J Hu Z Kaplan JM. HBL-1 patterns synaptic remodeling in *C. elegans*. Neuron (2012) 73:453–65. 10.1016/j.neuron.2011.11.025 22325199 PMC3278716

[46] Simon DJ Madison JM Conery AL Thompson-Peer KL Soskis M Ruvkun GB The microRNA miR-1 regulates a MEF-2-dependent retrograde signal at neuromuscular junctions. Cell (2008) 133:903–15. 10.1016/j.cell.2008.04.035 18510933 PMC2553566

[47] Hu Z Hom S Kudze T Tong XJ Choi S Aramuni G Neurexin and neuroligin mediate retrograde synaptic inhibition in *C. elegans*. Science (2012) 337:980–4. 10.1126/science.1224896 22859820 PMC3791080

[48] Persico AM Napolioni V. Autism genetics. Behav Brain Res (2013) 251:95–112. 10.1016/j.bbr.2013.06.012 23769996

[49] Li S Wang X Gu Y Chen C Wang Y Liu J Let-7 microRNAs regenerate peripheral nerve regeneration by targeting nerve growth factor. Mol Ther (2015) 23:423–33. 10.1038/mt.2014.220 25394845 PMC4351454

[50] Boulias K Horvitz HR. The *C. elegans* microRNA mir-71 acts in neurons to promote germline-mediated longevity through regulation of DAF-16/FOXO. Cell Metab (2012) 15:439–50. 10.1016/j.cmet.2012.02.014 22482727 PMC3344382

[51] Lin K Hsin H Libina N Kenyon C. Regulation of the *Caenorhabditis elegans* longevity protein DAF-16 by insulin/IGF-1 and germline signaling. Nat Genet (2001) 28:139–45. 10.1038/88850 11381260

[52] Libina N Berman JR Kenyon C. Tissue-specific activities of *C. elegans* DAF-16 in the regulation of lifespan. Cell (2003) 115:489–502. 10.1016/S0092-8674(03)00889-4 14622602

[53] Chawla G Sokol NS. MicroRNAs in Drosophila development. Int Rev Cell Mol Biol (2011) 286:1–65. 10.1016/B978-0-12-385859-7.00001-X 21199779

[54] Makeyev EV Zhang J Carrasco MA Maniatis T. The MicroRNA miR-124 promotes neuronal differentiation by triggering brain-specific alternative pre-mRNA splicing. Mol Cell (2007) 27:435–48. 10.1016/j.molcel.2007.07.015 17679093 PMC3139456

[55] Cheng LC Pastrana E Tavazoie M Doetsch F. miR-124 regulates adult neurogenesis in the subventricular zone stem cell niche. Nat Neurosci (2009) 12:399–408. 10.1038/nn.2294 19287386 PMC2766245

[56] Maiorano NA Mallamaci A. Promotion of embryonic cortico-cerebral neuronogenesis by miR-124. Neural Dev (2009) 4:40. 10.1186/1749-8104-4-40 19883498 PMC2777883

[57] Liu K Liu Y Mo W Qiu R Wang X Wu JY MiR-124 regulates early neurogenesis in the optic vesicle and forebrain, targeting NeuroD1. Nucleic Acids Res (2011) 39:2869–79. 10.1093/nar/gkq904 21131276 PMC3074159

[58] Weng R Cohen SM. Drosophila miR-124 regulates neuroblast proliferation through its target anachronism. Development (2012) 139:1427–34. 10.1242/dev.075143 22378639

[59] Sun K Westholm JO Tsurudome K Hagen JW Lu Y Kohwi M Neurophysiological defects and neuronal gene deregulation in Drosophila mir-124 mutants. PLoS Genet (2012) 8:e1002515. 10.1371/journal.pgen.1002515 22347817 PMC3276548

[60] Morante J Vallejo DM Desplan C Dominguez M. Conserved miR-8/miR-200 defines a glial niche that controls neuroepithelial expansion and neuroblast transition. Dev Cell (2013) 27:174–87. 10.1016/j.devcel.2013.09.018 24139822 PMC3931912

[61] Yuva-Aydemir Y Xu XL Aydemir O Gascon E Sayin S Zhou W Downregulation of the host gene jigr1 by miR-92 is essential for neuroblast self-renewal in Drosophila. PLoS Genet (2015) 11:e1005264. 10.1371/journal.pgen.1005264 26000445 PMC4441384

[62] Berdnik D Fan AP Potter CJ Luo L. MicroRNA processing pathway regulates olfactory neuron morphogenesis. Curr Biol (2008) 18:1754–9. 10.1016/j.cub.2008.09.045 19013069 PMC2612040

[63] Lee T Lee A Luo L. Development of the Drosophila mushroom bodies: sequential generation of three distinct types of neurons from a neuroblast. Development (1999) 126:4065–76. 10457015 10.1242/dev.126.18.4065

[64] Zars T Fischer M Schulz R Heisenberg M. Localization of a short-term memory in Drosophila. Science (2000) 288:672–5. 10.1126/science.288.5466.672 10784450

[65] Zhu S Chiang AS Lee T. Development of the Drosophila mushroom bodies: elaboration, remodeling and spatial organization of dendrites in the calyx. Development (2003) 130:2603–10. 10.1242/dev.00466 12736205

[66] Zhu S Lin S Kao CF Awasaki T Chiang AS Lee T. Gradients of the Drosophila Chinmo BTB-zinc finger protein govern neuronal temporal identity. Cell (2006) 127:409–22. 10.1016/j.cell.2006.08.045 17055440

[67] Wu YC Chen CH Mercer A Sokol NS. Let-7-complex microRNAs regulate the temporal identity of Drosophila mushroom body neurons via chinmo. Dev Cell (2012) 23:202–9. 10.1016/j.devcel.2012.05.013 22814608 PMC3401410

[68] Chawla G Sokol NS. Hormonal activation of let-7-C microRNAs via EcR is required for adult *Drosophila melanogaster* morphology and function. Development (2012) 139:1788–97. 10.1242/dev.077743 22510985 PMC3328179

[69] Parrish JZ Xu P Kim CC Jan LY Jan YN. The microRNA bantam functions in epithelial cells to regulate scaling growth of dendrite arbors in drosophila sensory neurons. Neuron (2009) 63:788–802. 10.1016/j.neuron.2009.08.006 19778508 PMC2772869

[70] Jiang N Soba P Parker E Kim CC Parrish JZ. The microRNA bantam regulates a developmental transition in epithelial cells that restricts sensory dendrite growth. Development (2014) 141:2657–68. 10.1242/dev.107573 24924190 PMC4067962

[71] Wang Y Wang H Li X Li Y. Epithelial microRNA-9a regulates dendrite growth through Fmi-Gq signaling in Drosophila sensory neurons. Dev Neurobiol (2015). 10.1002/dneu.22309 26016469

[72] Nolo R Abbott LA Bellen HJ. Senseless, a Zn finger transcription factor, is necessary and sufficient for sensory organ development in Drosophila. Cell (2000) 102:349–62. 10.1016/S0092-8674(00)00040-4 10975525

[73] Jafar-Nejad H Acar M Nolo R Lacin H Pan H Parkhurst SM Senseless acts as a binary switch during sensory organ precursor selection. Genes Dev (2003) 17:2966–78. 10.1101/gad.1122403 14665671 PMC289154

[74] Li X Cassidy JJ Reinke CA Fischboeck S Carthew RW. A microRNA imparts robustness against environmental fluctuation during development. Cell (2009) 137:273–82. 10.1016/j.cell.2009.01.058 19379693 PMC2674871

[75] Li Z Lu Y Xu XL Gao FB. The FTD/ALS-associated RNA-binding protein TDP-43 regulates the robustness of neuronal specification through microRNA-9a in Drosophila. Hum Mol Genet (2013) 22:218–25. 10.1093/hmg/dds420 23042786 PMC3526156

[76] Nesler KR Sand RI Symmes BA Pradhan SJ Boin NG Laun AE The miRNA pathway controls rapid changes in activity-dependent synaptic structure at the Drosophila melanogaster neuromuscular junction. PLoS One (2013) 8:e68385. 10.1371/journal.pone.0068385 23844193 PMC3699548

[77] Lu CS Zhai B Mauss A Landgraf M Gygi S Van Vactor D. MicroRNA-8 promotes robust motor axon targeting by coordinate regulation of cell adhesion molecules during synapse development. Philos Trans R Soc Lond B Biol Sci (2014) 369. 10.1098/rstb.2013.0517 25135978 PMC4142038

[78] Karres JS Hilgers V Carrera I Treisman J Cohen SM. The conserved microRNA miR-8 tunes atrophin levels to prevent neurodegeneration in Drosophila. Cell (2007) 131:136–45. 10.1016/j.cell.2007.09.020 17923093

[79] Pratt KG Khakhalin AS. Modeling human neurodevelopmental disorders in the Xenopus tadpole: from mechanisms to therapeutic targets. Dis Model Mech (2013) 6:1057–65. 10.1242/dmm.012138 23929939 PMC3759326

[80] Qiu R Liu K Liu Y Mo W Flynt AS Patton JG The role of miR-124a in early development of the Xenopus eye. Mech Dev (2009) 126:804–16. 10.1016/j.mod.2009.08.002 19703558 PMC4445405

[81] Baudet ML Zivraj KH Abreu-Goodger C Muldal A Armisen J Blenkiron C miR-124 acts through CoREST to control onset of Sema3A sensitivity in navigating retinal growth cones. Nat Neurosci (2012) 15:29–38. 10.1038/nn.2979 22138647 PMC3661270

[82] Decembrini S Bressan D Vignali R Pitto L Mariotti S Rainaldi G MicroRNAs couple cell fate and developmental timing in retina. Proc Natl Acad Sci U S A (2009) 106:21179–84. 10.1073/pnas.0909167106 19965369 PMC2781736

[83] Bonev B Pisco A Papalopulu N. MicroRNA-9 reveals regional diversity of neural progenitors along the anterior-posterior axis. Dev Cell (2011) 20:19–32. 10.1016/j.devcel.2010.11.018 21238922 PMC3361082

[84] Tarpey PS Raymond FL Nguyen LS Rodriguez J Hackett A Vandeleur L Mutations in UPF3B, a member of the nonsense-mediated mRNA decay complex, cause syndromic and nonsyndromic mental retardation. Nat Genet (2007) 39:1127–33. 10.1038/ng2100 17704778 PMC2872770

[85] Addington AM Gauthier J Piton A Hamdan FF Raymond A Gogtay N A novel frameshift mutation in UPF3B identified in brothers affected with childhood onset schizophrenia and autism spectrum disorders. Mol Psychiatry (2011) 16:238–9. 10.1038/mp.2010.59 20479756 PMC3024438

[86] Bruno IG Karam R Huang L Bhardwaj A Lou CH Shum EY Identification of a microRNA that activates gene expression by repressing nonsense-mediated RNA decay. Mol Cell (2011) 42:500–10. 10.1016/j.molcel.2011.04.018 21596314 PMC3123134

[87] Giraldez AJ Cinalli RM Glasner ME Enright AJ Thomson MJ Baskerville S MicroRNAs regulate brain morphogenesis in Zebrafish. Science (2005) 308:833–8. 10.1126/science.1109020 15774722

[88] Kapsimali M Kloosterman WP De Bruijn E Rosa F Plasterk RH Wilson SW. MicroRNAs show a wide diversity of expression profiles in the developing and mature central nervous system. Genome Biol (2007) 8:R173. 10.1186/gb-2007-8-8-r173 17711588 PMC2375003

[89] Ouchi Y Yamamoto J Iwamoto T. The heterochronic genes lin-28a and lin-28b play an essential and evolutionarily conserved role in early zebrafish development. PLoS One (2014) 9:e88086. 10.1371/journal.pone.0088086 24516585 PMC3916362

[90] Ristori E Lopez-Ramirez MA Narayanan A Hill-Teran G Moro A Calvo CF A Dicer-miR-107 interaction regulates biogenesis of specific miRNAs crucial for neurogenesis. Dev Cell (2015) 32:546–60. 10.1016/j.devcel.2014.12.013 25662174 PMC8950125

[91] Leucht C Stigloher C Wizenmann A Klafke R Folchert A Bally-Cuif L. MicroRNA-9 directs late organizer activity of the midbrain-hindbrain boundary. Nat Neurosci (2008) 11:641–8. 10.1038/nn.2115 18454145

[92] Coolen M Thieffry D Drivenes O Becker TS Bally-Cuif L. miR-9 controls the timing of neurogenesis through the direct inhibition of antagonistic factors. Dev Cell (2012) 22:1052–64. 10.1016/j.devcel.2012.03.003 22595676

[93] Coolen M Katz S Bally-Cuif L. miR-9: a versatile regulator of neurogenesis. Front Cell Neurosci (2013) 7:220. 10.3389/fncel.2013.00220 24312010 PMC3834235

[94] Koch P Lohr HB Driever W. A mutation in cnot8, component of the Ccr4-not complex regulating transcript stability, affects expression levels of developmental regulators and reveals a role of Fgf3 in development of caudal hypothalamic dopaminergic neurons. PLoS One (2014) 9:e113829. 10.1371/journal.pone.0113829 25478689 PMC4257555

[95] Sanchez-Simon FM Zhang XX Loh HH Law PY Rodriguez RE. Morphine regulates dopaminergic neuron differentiation via miR-133b. Mol Pharmacol (2010) 78:935–42. 10.1124/mol.110.066837 20716624 PMC2981367

[96] Lewellis SW Nagelberg D Subedi A Staton A Leblanc M Giraldez A Precise SDF1-mediated cell guidance is achieved through ligand clearance and microRNA-mediated decay. J Cell Biol (2013) 200:337–55. 10.1083/jcb.201207099 23382464 PMC3563679

[97] Wei C Thatcher EJ Olena AF Cha DJ Perdigoto AL Marshall AF miR-153 regulates SNAP-25, synaptic transmission, and neuronal development. PLoS One (2013) 8:e57080. 10.1371/journal.pone.0057080 23451149 PMC3581580

[98] Liu J Carmell MA Rivas FV Marsden CG Thomson JM Song JJ Argonaute2 is the catalytic engine of mammalian RNAi. Science (2004) 305:1437–41. 10.1126/science.1102513 15284456

[99] Babiarz JE Hsu R Melton C Thomas M Ullian EM Blelloch R. A role for noncanonical microRNAs in the mammalian brain revealed by phenotypic differences in Dgcr8 versus Dicer1 knockouts and small RNA sequencing. RNA (2011) 17:1489–501. 10.1261/rna.2442211 21712401 PMC3153973

[100] Meechan DW Maynard TM Tucker ES Lamantia AS. Three phases of DiGeorge/22q11 deletion syndrome pathogenesis during brain development: patterning, proliferation, and mitochondrial functions of 22q11 genes. Int J Dev Neurosci (2011) 29:283–94. 10.1016/j.ijdevneu.2010.08.005 20833244 PMC3770287

[101] Grishok A Pasquinelli AE Conte D Li N Parrish S Ha I Genes and mechanisms related to RNA interference regulate expression of the small temporal RNAs that control *C. elegans* developmental timing. Cell (2001) 106:23–34. 10.1016/S0092-8674(01)00431-7 11461699

[102] Lee YS Nakahara K Pham JW Kim K He Z Sontheimer EJ Distinct roles for Drosophila Dicer-1 and Dicer-2 in the siRNA/miRNA silencing pathways. Cell (2004) 117:69–81. 10.1016/S0092-8674(04)00261-2 15066283

[103] Davis TH Cuellar TL Koch SM Barker AJ Harfe BD Mcmanus MT Conditional loss of Dicer disrupts cellular and tissue morphogenesis in the cortex and hippocampus. J Neurosci (2008) 28:4322–30. 10.1523/JNEUROSCI.4815-07.2008 18434510 PMC3844796

[104] Tao J Wu H Lin Q Wei W Lu XH Cantle JP Deletion of astroglial Dicer causes non-cell-autonomous neuronal dysfunction and degeneration. J Neurosci (2011) 31:8306–19. 10.1523/JNEUROSCI.0567-11.2011 21632951 PMC3500097

[105] Kawase-Koga Y Otaegi G Sun T. Different timings of Dicer deletion affect neurogenesis and gliogenesis in the developing mouse central nervous system. Dev Dyn (2009) 238:2800–12. 10.1002/dvdy.22109 19806666 PMC2831750

[106] Barca-Mayo O De Pietri Tonelli D. Convergent microRNA actions coordinate neocortical development. Cell Mol Life Sci (2014) 71:2975–95. 10.1007/s00018-014-1576-5 24519472 PMC4111863

[107] Nowakowski TJ Mysiak KS Pratt T Price DJ. Functional dicer is necessary for appropriate specification of radial glia during early development of mouse telencephalon. PLoS One (2011) 6:e23013. 10.1371/journal.pone.0023013 21826226 PMC3149632

[108] Huang T Liu Y Huang M Zhao X Cheng L. Wnt1-cre-mediated conditional loss of Dicer results in malformation of the midbrain and cerebellum and failure of neural crest and dopaminergic differentiation in mice. J Mol Cell Biol (2010) 2:152–63. 10.1093/jmcb/mjq008 20457670

[109] Zehir A Hua LL Maska EL Morikawa Y Cserjesi P. Dicer is required for survival of differentiating neural crest cells. Dev Biol (2010) 340:459–67. 10.1016/j.ydbio.2010.01.039 20144605 PMC2878775

[110] Schwamborn JC Berezikov E Knoblich JA. The TRIM-NHL protein TRIM32 activates microRNAs and prevents self-renewal in mouse neural progenitors. Cell (2009) 136:913–25. 10.1016/j.cell.2008.12.024 19269368 PMC2988196

[111] Cimadamore F Amador-Arjona A Chen C Huang CT Terskikh AV. SOX2-LIN28/let-7 pathway regulates proliferation and neurogenesis in neural precursors. Proc Natl Acad Sci U S A (2013) 110:E3017–26. 10.1073/pnas.1220176110 23884650 PMC3740872

[112] Zhao C Sun G Li S Lang MF Yang S Li W MicroRNA let-7b regulates neural stem cell proliferation and differentiation by targeting nuclear receptor TLX signaling. Proc Natl Acad Sci U S A (2010) 107:1876–81. 10.1073/pnas.0908750107 20133835 PMC2836616

[113] Tan SL Ohtsuka T Gonzalez A Kageyama R. MicroRNA9 regulates neural stem cell differentiation by controlling Hes1 expression dynamics in the developing brain. Genes Cells (2012) 17:952–61. 10.1111/gtc.12009 23134481

[114] Shibata M Nakao H Kiyonari H Abe T Aizawa S. MicroRNA-9 regulates neurogenesis in mouse telencephalon by targeting multiple transcription factors. J Neurosci (2011) 31:3407–22. 10.1523/JNEUROSCI.5085-10.2011 21368052 PMC6623912

[115] Delaloy C Liu L Lee JA Su H Shen F Yang GY MicroRNA-9 coordinates proliferation and migration of human embryonic stem cell-derived neural progenitors. Cell Stem Cell (2010) 6:323–35. 10.1016/j.stem.2010.02.015 20362537 PMC2851637

[116] Akerblom M Sachdeva R Barde I Verp S Gentner B Trono D MicroRNA-124 is a subventricular zone neuronal fate determinant. J Neurosci (2012) 32:8879–89. 10.1523/JNEUROSCI.0558-12.2012 22745489 PMC4434222

[117] Conaco C Otto S Han JJ Mandel G. Reciprocal actions of REST and a microRNA promote neuronal identity. Proc Natl Acad Sci U S A (2006) 103:2422–7. 10.1073/pnas.0511041103 16461918 PMC1413753

[118] Wu J Xie X. Comparative sequence analysis reveals an intricate network among REST, CREB and miRNA in mediating neuronal gene expression. Genome Biol (2006) 7:R85. 10.1186/gb-2006-7-9-r85 17002790 PMC1794552

[119] Bassani S Zapata J Gerosa L Moretto E Murru L Passafaro M. The neurobiology of X-linked intellectual disability. Neuroscientist (2013) 19:541–52. 10.1177/1073858413493972 23820068

[120] Rosti RO Sadek AA Vaux KK Gleeson JG. The genetic landscape of autism spectrum disorders. Dev Med Child Neurol (2014) 56:12–8. 10.1111/dmcn.12278 24116704

[121] Yoo AS Sun AX Li L Shcheglovitov A Portmann T Li Y MicroRNA-mediated conversion of human fibroblasts to neurons. Nature (2011) 476:228–31. 10.1038/nature10323 21753754 PMC3348862

[122] Vierbuchen T Ostermeier A Pang ZP Kokubu Y Sudhof TC Wernig M. Direct conversion of fibroblasts to functional neurons by defined factors. Nature (2010) 463:1035–41. 10.1038/nature08797 20107439 PMC2829121

[123] Gong X Zhang K Wang Y Wang J Cui Y Li S MicroRNA-130b targets Fmr1 and regulates embryonic neural progenitor cell proliferation and differentiation. Biochem Biophys Res Commun (2013) 439:493–500. 10.1016/j.bbrc.2013.08.096 24021279

[124] Anderegg A Lin HP Chen JA Caronia-Brown G Cherepanova N Yun B An Lmx1b-miR135a2 regulatory circuit modulates Wnt1/Wnt signaling and determines the size of the midbrain dopaminergic progenitor pool. PLoS Genet (2013) 9:e1003973. 10.1371/journal.pgen.1003973 24348261 PMC3861205

[125] Gaughwin P Ciesla M Yang H Lim B Brundin P. Stage-specific modulation of cortical neuronal development by Mmu-miR-134. Cereb Cortex (2011) 21:1857–69. 10.1093/cercor/bhq262 21228099

[126] Chang SJ Weng SL Hsieh JY Wang TY Chang MD Wang HW. MicroRNA-34a modulates genes involved in cellular motility and oxidative phosphorylation in neural precursors derived from human umbilical cord mesenchymal stem cells. BMC Med Genomics (2011) 4:65. 10.1186/1755-8794-4-65 21923954 PMC3195087

[127] Clovis YM Enard W Marinaro F Huttner WB De Pietri Tonelli D. Convergent repression of Foxp2 3’UTR by miR-9 and miR-132 in embryonic mouse neocortex: implications for radial migration of neurons. Development (2012) 139:3332–42. 10.1242/dev.078063 22874921

[128] Volvert ML Prevot PP Close P Laguesse S Pirotte S Hemphill J MicroRNA targeting of CoREST controls polarization of migrating cortical neurons. Cell Rep (2014) 7:1168–83. 10.1016/j.celrep.2014.03.075 24794437

[129] Rago L Beattie R Taylor V Winter J. miR379-410 cluster miRNAs regulate neurogenesis and neuronal migration by fine-tuning N-cadherin. EMBO J (2014) 33:906–20. 10.1002/embj.201386591 24614228 PMC4194114

[130] Franzoni E Booker SA Parthasarathy S Rehfeld F Grosser S Srivatsa S miR-128 regulates neuronal migration, outgrowth and intrinsic excitability via the intellectual disability gene Phf6. Elife (2015) 4. 10.7554/eLife.04263 25556700 PMC4337614

[131] Valluy J Bicker S Aksoy-Aksel A Lackinger M Sumer S Fiore R A coding-independent function of an alternative Ube3a transcript during neuronal development. Nat Neurosci (2015) 18:666–73. 10.1038/nn.3996 25867122

[132] Magill ST Cambronne XA Luikart BW Lioy DT Leighton BH Westbrook GL microRNA-132 regulates dendritic growth and arborization of newborn neurons in the adult hippocampus. Proc Natl Acad Sci U S A (2010) 107:20382–7. 10.1073/pnas.1015691107 21059906 PMC2996687

[133] Pathania M Torres-Reveron J Yan L Kimura T Lin TV Gordon V miR-132 enhances dendritic morphogenesis, spine density, synaptic integration, and survival of newborn olfactory bulb neurons. PLoS One (2012) 7:e38174. 10.1371/journal.pone.0038174 22693596 PMC3364964

[134] Yang D Li T Wang Y Tang Y Cui H Tang Y miR-132 regulates the differentiation of dopamine neurons by directly targeting Nurr1 expression. J Cell Sci (2012) 125:1673–82. 10.1242/jcs.086421 22328530

[135] Franke K Otto W Johannes S Baumgart J Nitsch R Schumacher S. miR-124-regulated RhoG reduces neuronal process complexity via ELMO/Dock180/Rac1 and Cdc42 signalling. EMBO J (2012) 31:2908–21. 10.1038/emboj.2012.130 22588079 PMC3395090

[136] Siegert S Seo J Kwon EJ Rudenko A Cho S Wang W The schizophrenia risk gene product miR-137 alters presynaptic plasticity. Nat Neurosci (2015) 18:1008–16. 10.1038/nn.4023 26005852 PMC4506960

[137] Nowak JS Michlewski G. miRNAs in development and pathogenesis of the nervous system. Biochem Soc Trans (2013) 41:815–20. 10.1042/BST20130044 23863137

[138] Sempere LF Freemantle S Pitha-Rowe I Moss E Dmitrovsky E Ambros V. Expression profiling of mammalian microRNAs uncovers a subset of brain-expressed microRNAs with possible roles in murine and human neuronal differentiation. Genome Biol (2004) 5:R13. 10.1186/gb-2004-5-8-p13 15003116 PMC395763

[139] Ziats MN Rennert OM. Identification of differentially expressed microRNAs across the developing human brain. Mol Psychiatry (2014) 19:848–52. 10.1038/mp.2013.93 23917947 PMC3840150

[140] Fineberg SK Kosik KS Davidson BL. MicroRNAs potentiate neural development. Neuron (2009) 64:303–9. 10.1016/j.neuron.2009.10.020 19914179

[141] Adlakha YK Saini N. Brain microRNAs and insights into biological functions and therapeutic potential of brain enriched miRNA-128. Mol Cancer (2014) 13:33. 10.1186/1476-4598-13-33 24555688 PMC3936914

[142] Roese-Koerner B Stappert L Koch P Brustle O Borghese L. Pluripotent stem cell-derived somatic stem cells as tool to study the role of microRNAs in early human neural development. Curr Mol Med (2013) 13:707–22. 10.2174/1566524011313050003 23642053

[143] Im HI Kenny PJ. MicroRNAs in neuronal function and dysfunction. Trends Neurosci (2012) 35:325–34. 10.1016/j.tins.2012.01.004 22436491 PMC3565236

[144] Banerjee-Basu S Larsen E Muend S Common microRNAs target established ASD genes. Front Neurol (2014) 5:205 10.3389/fneur.2014.00205 25389413 PMC4211397

[145] Sun E Shi Y. MicroRNAs: Small molecules with big roles in neurodevelopment and diseases. Exp Neurol (2015) 268:46–53. 10.1016/j.expneurol.2014.08.005 25128264

[146] Perkins DO Jeffries CD Jarskog LF Thomson JM Woods K Newman MA microRNA expression in the prefrontal cortex of individuals with schizophrenia and schizoaffective disorder. Genome Biol (2007) 8:R27. 10.1186/gb-2007-8-2-r27 17326821 PMC1852419

[147] Moreau MP Bruse SE David-Rus R Buyske S Brzustowicz LM. Altered microRNA expression profiles in postmortem brain samples from individuals with schizophrenia and bipolar disorder. Biol Psychiatry (2011) 69:188–93. 10.1016/j.biopsych.2010.09.039 21183010 PMC3038345

[148] Krystal JH Karper LP Seibyl JP Freeman GK Delaney R Bremner JD Subanesthetic effects of the noncompetitive NMDA antagonist, ketamine, in humans. Psychotomimetic, perceptual, cognitive, and neuroendocrine responses. Arch Gen Psychiatry (1994) 51:199–214. 10.1001/archpsyc.1994.03950030035004 8122957

[149] Newcomer JW Farber NB Jevtovic-Todorovic V Selke G Melson AK Hershey T Ketamine-induced NMDA receptor hypofunction as a model of memory impairment and psychosis. Neuropsychopharmacology (1999) 20:106–18. 10.1016/S0893-133X(98)00067-0 9885791

[150] Stone JM Erlandsson K Arstad E Squassante L Teneggi V Bressan RA Relationship between ketamine-induced psychotic symptoms and NMDA receptor occupancy: a [(123)I]CNS-1261 SPET study. Psychopharmacology (Berl) (2008) 197:401–8. 10.1007/s00213-007-1047-x 18176855

[151] Cheng HY Papp JW Varlamova O Dziema H Russell B Curfman JP microRNA modulation of circadian-clock period and entrainment. Neuron (2007) 54:813–29. 10.1016/j.neuron.2007.05.017 17553428 PMC2590749

[152] Miller BH Zeier Z Xi L Lanz TA Deng S Strathmann J MicroRNA-132 dysregulation in schizophrenia has implications for both neurodevelopment and adult brain function. Proc Natl Acad Sci U S A (2012) 109:3125–30. 10.1073/pnas.1113793109 22315408 PMC3286960

[153] Ripke S Sanders AR Kendler KS Levinson DF Sklar P Holmans PA Genome-wide association study identifies five new schizophrenia loci. Nat Genet (2011) 43:969–76. 10.1038/ng.940 21926974 PMC3303194

[154] Whalley HC Papmeyer M Romaniuk L Sprooten E Johnstone EC Hall J Impact of a microRNA MIR137 susceptibility variant on brain function in people at high genetic risk of schizophrenia or bipolar disorder. Neuropsychopharmacology (2012) 37:2720–9. 10.1038/npp.2012.137 22850735 PMC3473338

[155] Mothersill O Morris DW Kelly S Rose EJ Fahey C O’brien C Effects of MIR137 on fronto-amygdala functional connectivity. Neuroimage (2014) 90:189–95. 10.1016/j.neuroimage.2013.12.019 24361663

[156] Kuswanto CN Sum MY Qiu A Sitoh YY Liu J Sim K. The impact of genome wide supported microRNA-137 (MIR137) risk variants on frontal and striatal white matter integrity, neurocognitive functioning, and negative symptoms in schizophrenia. Am J Med Genet B Neuropsychiatr Genet (2015) 168:317–26. 10.1002/ajmg.b.32314 25921703

[157] Siew WH Tan KL Babaei MA Cheah PS Ling KH. MicroRNAs and intellectual disability (ID) in Down syndrome, X-linked ID, and Fragile X syndrome. Front Cell Neurosci (2013) 7:41. 10.3389/fncel.2013.00041 23596395 PMC3625835

[158] Verkerk AJ Pieretti M Sutcliffe JS Fu YH Kuhl DP Pizzuti A Identification of a gene (FMR-1) containing a CGG repeat coincident with a breakpoint cluster region exhibiting length variation in fragile X syndrome. Cell (1991) 65:905–14. 10.1016/0092-8674(91)90397-H 1710175

[159] Ishizuka A Siomi MC Siomi H. A Drosophila fragile X protein interacts with components of RNAi and ribosomal proteins. Genes Dev (2002) 16:2497–508. 10.1101/gad.1022002 12368261 PMC187455

[160] Amir RE Van Den Veyver IB Wan M Tran CQ Francke U Zoghbi HY. Rett syndrome is caused by mutations in X-linked MECP2, encoding methyl-CpG-binding protein 2. Nat Genet (1999) 23:185–8. 10.1038/13810 10508514

[161] Cheng TL Wang Z Liao Q Zhu Y Zhou WH Xu W MeCP2 suppresses nuclear microRNA processing and dendritic growth by regulating the DGCR8/Drosha complex. Dev Cell (2014) 28:547–60. 10.1016/j.devcel.2014.01.032 24636259

[162] Abu-Elneel K Liu T Gazzaniga FS Nishimura Y Wall DP Geschwind DH Heterogeneous dysregulation of microRNAs across the autism spectrum. Neurogenetics (2008) 9:153–61. 10.1007/s10048-008-0133-5 18563458

[163] Durand CM Betancur C Boeckers TM Bockmann J Chaste P Fauchereau F Mutations in the gene encoding the synaptic scaffolding protein SHANK3 are associated with autism spectrum disorders. Nat Genet (2007) 39:25–7. 10.1038/ng1933 17173049 PMC2082049

[164] Duong L Klitten LL Moller RS Ingason A Jakobsen KD Skjodt C Mutations in NRXN1 in a family multiply affected with brain disorders: NRXN1 mutations and brain disorders. Am J Med Genet B Neuropsychiatr Genet (2012) 159B:354–8. 10.1002/ajmg.b.32036 22337556

[165] Mundalil Vasu M Anitha A Thanseem I Suzuki K Yamada K Takahashi T Serum microRNA profiles in children with autism. Mol Autism (2014) 5:40. 10.1186/2040-2392-5-40 25126405 PMC4132421

[166] Huang F Long Z Chen Z Li J Hu Z Qiu R Investigation of gene regulatory networks associated with autism spectrum disorder based on MiRNA expression in China. PLoS One (2015) 10:e0129052. 10.1371/journal.pone.0129052 26061495 PMC4462583

